# Enhanced Generation of Human Induced Pluripotent Stem Cells from Peripheral Blood and Using Their Mesoderm Differentiation Ability to Regenerate Infarcted Myocardium

**DOI:** 10.1155/2022/4104622

**Published:** 2022-02-11

**Authors:** Ju-Young Kim, Han-Mo Yang, Joo-Eun Lee, Mika Jeon, Sang-Bum Bang, Jihye You, Joonoh Kim, Jaewon Lee, Jin Hur, Hyo-Soo Kim

**Affiliations:** ^1^New Drug Development Division, K-BioCELF Inc., Suwon, Gyeonggi-do, Republic of Korea; ^2^Cardiovascular Center and Department of Internal Medicine, Seoul National University Hospital, Seoul, Republic of Korea; ^3^Department of Convergence Medicine, Pusan National University School of Medicine, Yangsan, Gyeongsangnam-do, Republic of Korea

## Abstract

Тhe most pressing issue in generating induced pluripotent stem cells (iPSCs) in clinical practice is the cell source. Compared to human dermal fibroblasts (HDFs), which have been widely used, human peripheral blood could be a more easily obtainable alternative. However, iPSCs generated from fresh peripheral blood require inconvenient specific methods including isolation. Recently, we succeeded in isolating and culturing human heart-derived circulating cells called circulating multipotent stem (CiMS) cells. Here, we investigated the generation efficiency of CiMS-derived iPSCs (CiMS-iPSCs) and tested their differentiation potential into mesodermal lineages and cardiovascular cells. We isolated and cultured CiMS cells from peripheral mononuclear cells with a high efficiency. Moreover, our method succeeded in reprogramming the CiMS cells and generating iPSCs with higher efficiency compared to when HDFs were used. Compared to HDF-iPSCs or human embryonic stem cells (hESCs), CiMS-iPSCs showed high differentiation potential into mesodermal lineage cells and subsequently into endothelial cells, vascular smooth muscle cells, and cardiomyocytes. Further, we checked the epigenetic status of each cell type. While methylation of the CpG site of the brachyury T promoter did not differ between cell types, the histone H3 lysine 4 trimethylation level in the brachyury T promoter region was enhanced in CiMS-iPSCs, compared to that in other cell types. In contrast, histone H3 lysine 9 acetylation was downregulated during the differentiation process of the CiMS-iPSCs. In the myocardial infarction model, the CiMS-iPSCs group showed more therapeutic potential in regenerating the myocardium than other cell types. Our study showed a new method to isolate human heart-derived stem cells from human peripheral blood and to generate iPSCs efficiently. Due to epigenetic memory, these CiMS-iPSCs easily differentiated into cardiovascular lineage cells, resulting in improved efficiency *in vivo*. These results suggest that our new method using CiMS cells has therapeutic potential in regenerative medicine using cell therapy.

## 1. Introduction

In 2006, Takahashi and Yamanaka [1] found that ectopic expression of Oct3/4, Sox2, Klf4, and c-Myc using viral (retroviral) gene transfer can transform murine somatic cells into induced pluripotent stem cells (iPSCs). One year later, human iPSCs from fibroblasts were independently generated by two research groups [2, 3]. Since iPSCs have similar pluripotent potential to embryonic stem (ES) cells, they can differentiate into almost every somatic cell type. Furthermore, they can be produced from autologous sources without ethical concerns and the problem of immune rejection, unlike that of ES cells. Therefore, they have been considered ideal for patient- and disease-specific regenerative therapy.

In humans, fibroblasts are typically used for reprogramming. However, because of the invasive preparation method and the need for a long culture period for application in reprogramming [2], several new candidates have been proposed. Since then, human terminally differentiated circulating T cells, which are highly accessible and can be obtained noninvasively, have been isolated and used for reprogramming [3]. However, these cells showed low virus transduction efficiency and T cell receptor gene rearrangement patterns in the original patient T cell clone.

Recently, we succeeded in culturing a new type of adult stem cells isolated from human peripheral blood samples for reprogramming of donor cells. These are multipotent stem cells derived from the heart endocardium called circulating multipotent stem (CiMS) cells [4]. CiMS cells are advantageous for iPSC production, because they divide actively enough to store extra cells and have high transduction efficiency. In addition, CiMS can be obtained from only 10 ml of blood, which can be obtained in the outpatient department.

Selecting donor cells for reprogramming is also important, because some epigenetic characteristics of the original cell can remain and form the different characteristics of multiple iPSCs [5, 6]. Recent studies described that some epigenetic status of original cell can be remained and make differentiation potentials distinguishable among iPSCs [6, 7]. CiMS cells are multipotent stem cells derived from the heart endocardium. Moreover, we observed high expression of GATA4, an early cardiomyocyte (CMC) differentiation marker, and SOX17, which is involved in heart development, in CiMS cells [8]. Thus, we presumed that CiMS-derived iPSCs (CiMS-iPSCs) could show higher potency in differentiating into cardiovascular cells.

Loosely compacted chromatin is epigenetically more accessible. Typically, DNA methylation and histone modification can alter chromatin compaction level and genetic activity. DNA methylation at CpG islands makes chromatin more compact [9–11]. In general, histone H3 lysine 4 trimethylation (H3K4me3) is observed at gene promoters and is considered a genetically active signal [12]. In contrast, H3K27me3 and H3K9ac act as genetically repressive signals [13]. We presumed that CiMS could remain in an euchromatic state in the mesodermal gene [14, 15]. In this study, we examined the generation efficiency of CiMS-iPSCs and tested their differentiation potential into cardiovascular cells, compared to other cell types.

## 2. Materials and Methods

### 2.1. Ethics Statement

All human samples were obtained with written informed consent after the approval by the Institutional Review Board (IRB) of Seoul National University Hospital (Approval No. H-0908-036-290). All animal experiments were performed after receiving approval from the Institutional Animal Care and Use Committee (IACUC) of Clinical Research Institute in Seoul National University Hospital and complied with the National Research Council (NRC) “Guidelines for the Care and Use of Laboratory Animals.”

### 2.2. Isolation of Peripheral Blood Mononuclear Cell (PBMC) and Culture of CiMS

This study was approved by the Institutional Review Board of the Seoul National University Hospital (IRB Number). Human peripheral blood samples (10 cc) were obtained from donors after informed consent. PBMCs were isolated from the blood samples using Ficoll-Paque PLUS (GE Healthcare, NJ, USA) according to the manufacturer's recommendations. Freshly isolated PBMCs were suspended in EGM-2MV BulletKit™ (Lonza, Basel, Switzerland) and seeded on the fibronectin-coated (Sigma-Aldrich, MO, USA) six-well plate at 6 × 10^6^ cells per well. The media were changed every single day for up to 2 weeks after plating. Adherent CiMS cells were observed from as early as five days after the start of culture and gradually formed colonies. CiMS were passaged using 0.05% Trypsin-EDTA solution.

### 2.3. Infection of Yamanaka's Reprogramming Factor Retrovirus and Generation of Induced Pluripotent Stem Cells

Human embryonic kidney (HEK) 293T cells were plated and transfected with retroviral vectors containing human OCT3/4, SOX2, KLF4, c-Myc gene, and packaging vectors with PEI solution (Sigma-Aldrich). Forty-eight hours after transfection, the retrovirus-containing supernatant was harvested and concentrated using ultracentrifugation with 25,000 rpm for 1 hour 30 min in 4°C. CiMS cells were seeded at 2 × 10^5^ cells per well in a 6-well plate before transduction. Concentrated retroviruses encoding the four reprogramming factors were added to CiMS cells with the 10 *μ*g/ml Polybrene (Sigma-Aldrich). Twenty-four hours after transduction, the transduction medium was changed with new fresh EGM-2MV medium. Six days after transduction, transduced CiMS cells were harvested by trypsinization and replated at 2 × 10^5^ cells onto new mitomycin C- (MMC-) treated STO feeder layers in a 6-well plate. Two days later, the medium was changed with human ES cell medium supplemented with 10 ng/ml bFGF (R&D systems, MN, USA) and the medium was replaced every single day. Fourteen days after transduction, iPS cell colonies were mechanically picked and transferred into new MMC-treated STO feeder layers.

### 2.4. Real-Time PCR

Real-time PCR was performed using FastStart SYBR Green (Roche, Mannheim, Germany) and analyzed with Applied Biosystems 7500 Real-Time PCR System (Applied Biosystems). Gene expression level was normalized to the level of 18s rRNA and quantified using the 2^(-∆Ct)^ method. PCR primers are listed in Supplementary Table S1.

### 2.5. Gene Expression Profiling

Total RNA from human ES cells (hESCs), CiMS-iPSCs, and CIMS were isolated using RNeasy Mini Kit columns (Qiagen, Hagen, Germany). RNA samples hybridized to the GeneChip® Human Gene 1.0 ST arrays and the chips were stained and scanned using a GeneChip Array scanner 3000 7G (Affymetrix, CA, USA). The expression intensity data were extracted from the scanned images using Affymetrix Command Console software version 1.1 and analyzed using the Robust Multi-array Average (RMA) algorithm implemented in the Affymetrix Expression Console software (version 1.3.1.).

### 2.6. Teratoma Formation

The CiMS-iPSCs were harvested by 10 units/ml dispase (Roche) treatment for one hour at 37°C and collected into 15 ml tubes and centrifuged, and the pellets were suspended in DMEM/F12 mixed with Matrigel (BD Biosciences, NJ, USA). These harvested cells were injected subcutaneously into the dorsal flank of NOD SCID mouse (Charles River, MA, USA). Mice were sacrificed after 12 weeks according to tumor development. Teratomas were fixed with 4% paraformaldehyde in PBS for 48 hours and embedded in paraffin. The teratomas were sectioned and stained with hematoxylin and eosin.

### 2.7. Bisulfite PCR

One milligram of genomic DNA was isolated from human iPS cells and ES cells. Each DNA was treated with bisulfite using EpiTect Bisulfite Kit (Qiagen). To amplify the promoter regions, such as human Oct3/4 and NANOG or Brachury T, we used PCR. The PCR products were purified using Zymoclean gel DNA recovery kit (Zymo). The insert was subcloned into pCR2.1-TOPO vector (Invitrogen) and sequenced with M13 forward and reverse primers.

### 2.8. In Vitro Differentiation into Three Different Lineages

Each pluripotent stem cell (PSC) harvested by dispase (Invitrogen) treatment and seeded in Matrigel- (Corning) coated 35 mm dishes (Corning) at a density of 2 × 10^6^ cells in mTesR1 (Stemcell Technologies) medium with 10 *μ*M Y-27632. When each PSC was grown to near full confluency, differentiation induction was started. For early ectoderm differentiation, PSCs were cultured in KSR medium supplemented with 10 *μ*M SB431542 (TOCRIS) and 500 nM LDN193189 (Sigma) for 3 days. Every 24 hours, medium was changed. KSR medium is composed of DMEM containing 15% KnockOut serum replacement, 1% Pen Strep, 1% L-glutamine, 1% MEM non-essential amino acids, and 1% 2-mercaptoethanol. For early mesoderm differentiation, PSCs were cultured in RPMI1640 containing 1% B27 without insulin (Thermo Fisher Scientific) supplemented with 10 *μ*M CHIR99021 (Cayman) for 2 days. Every 24 hours, the medium was changed. For early endoderm differentiation, PSCs were treated with 100 ng/ml Activin A (Roche), 3 *μ*M CHIR99021 (Cayman) in RPMI 1640 containing 1x Glutamax I (Invitrogen) and 1% Pen Strep, and 2% fetal bovine serum (Invitrogen).

### 2.9. *In Vitro* Differentiation into Cardiovascular Cells

For cardiomyocyte differentiation, PSCs were treated with 10 *μ*M CHIR 99021 in RPMI1640 containing 1% B27 without insulin. The next day, cells were stimulated 20 ng/ml Activin A (R&D systems) for 24 hours and changed with RPMI1640 containing 1% B27 without insulin. The next day, cells were stimulated 5 *μ*M IWR1 (Sigma) in cardiomyocyte differentiation media for 48 hours without media change. And then, cells were maintained at cardiomyocyte medium for 2 days. And after 2 days, cells maintained in RPMI 1640 containing 1% B27. For endothelial cell differentiation, PSCs were treated with RPMI1640 containing 1% B27 and CHIR 99021. The next day, cells were stimulated with RPMI1640 containing 1% B27 medium with 100 ng/ml BMP4 (R&D systems) and 10 *μ*M PD98059 (Sigma). Subsequently, the cells were cultured in RPMI1640 containing 1% B27 medium with 10 *μ*M VEGF (Sigma) and 100 ng/ml bFGF 50 ng/ml for 20 days. For vascular smooth muscle cell differentiation, PSCs were cultured in RPMI 1640 containing 1% B27 and 10 *μ*M CHIR99021 for 1 day. The next day, the medium was changed with RPMI 1640 containing 1% B27 supplemented with 100 ng/ml BMP4 and 10 *μ*M LY290002 (PeproTech). And the third day of differentiation, the medium was changed with Endothelial Cell Growth Medium (Lonza) supplemented with 20 ng/ml PDGF BB (R&D systems) and 2 ng/ml TGF-*β*1 (PeproTech). For the reverse experiment, the H3K4 transferase (KMT) inhibitor, MM102, and H3K9 KMT inhibitor, chaetocin, were treated during cardiomyocyte differentiation to identify the effect of histone modification.

### 2.10. Immunofluorescent Staining for Marker of Differentiated Cardiovascular Cell

For immunofluorescent staining, cells were washed twice with PBS and fixed with 1% paraformaldehyde (PFA) or cold methanol for 10 minutes. After washing away the PFA or methanol with 0.05% TBS-T three times, blocking/permeabilization process was performed with 1%BSA solution including 0.05% Triton X-100. Cells were incubated with primary antibodies overnight at 4°C. Goat anti-PECAM-1 (M-20) (Santa Cruz Biotechnology), mouse anti-SMA (1A4) (Abcam), and monoclonal anti-*α* sarcomeric actin (Sigma-Aldrich) antibodies were used as primary antibodies. Alexa Flour 488 donkey anti-mouse IgG, Alexa Flour 555 donkey anti-goat IgG, and Alexa Flour 555 donkey anti-mouse IgM (Invitrogen) were used as secondary antibody. Nuclei were stained with DAPI (4′,6-diamidino-2-phenylindole). Samples were observed using a confocal microscope (Carl Zeiss).

### 2.11. Chromatin Immunoprecipitation Assay

Chromatin immunoprecipitation (ChIP) assay was performed using chromatin immunoprecipitation (ChIP) assay kit (Merck) and followed manufacturer's recommendation. 1 × 10^6^ cells were used for each immunoprecipitation reaction. Histone H3 tri methyl K4, Histone H3 acetyl K9, and Histone H3 tri methyl K27 antibodies from Abcam were used. For negative control of ChIP reaction, Rabbit IgG (Invitrogen) was used also included to allow for normalization. Primer information is listed in Table S1.

### 2.12. *In Vivo* Experiments

For heart injection, the stem cells of each group were differentiated to mesoderm progenitor cells. The cells of each group were treated with 10 *μ*M CHIR 99021 in RPMI1640 containing 1% B27 without insulin. After 24 hours, the medium containing CHIR99021 was changed to RPMI-B27 without insulin and incubated for additional 24 hours. Differentiated cells at this early mesodermal progenitor stage were harvested and prepared for injection into a mouse myocardial infarction (MI) model. For MI model, male nude mice (6–8 weeks old) were anesthetized by inhalation of 1.5% isoflurane. 5 *μ*l PBS containing a half million cells was injected into the myocardium of the mice with or without surgical manipulation such as the ligation of the coronary artery. The measurement of heart function with echocardiography was performed at baseline and after two weeks after myocardial infarction. Mouse heart tissues were harvested and fixed in 4% PFA overnight in room temperature. The tissues were embedded in paraffin and cut into 3 *μ*m thick sections. After deparaffination, all slides were boiled in retrieval solution (Dako). Goat anti-PECAM-1 (M-20) (Santa Cruz Biotechnology), mouse anti-SMA (1A4) (Abcam), and monoclonal anti-*α* sarcomeric actin (Sigma-Aldrich) antibodies were used as primary antibodies. Alexa Flour 488 donkey anti-mouse IgG, Alexa Flour 555 donkey anti-goat IgG, and Alexa Flour 555 donkey anti-mouse IgM (Invitrogen) were used as secondary antibodies. In each experiment, the controls were a medium- (PBS) injected group. The percentages of positive, a-SA-positive, SMA-positive, and CD31-positive cells versus total nucleated cells were quantified in 5 different sectors per tissue section in the peri-infarct zone at day 28 after the injection of cells.

### 2.13. Statistical Analysis

All data were calculated as mean ± SD. Group comparisons were performed by one-way ANOVA using GraphPad Prism 5 (GraphPad Software, San Diego, CA, USA), and the number of asterisks on top of each graphs means statistical significance; “∗,” “∗∗,” and “∗∗∗” mean that the *p* value range is 0.01 to 0.05, 0.001 to 0.01, and 0.001 to 0.01, respectively.

## 3. Results

### 3.1. High Reprogramming Efficiency of CiMS Cells from Human Peripheral Blood

**Figure 1:**
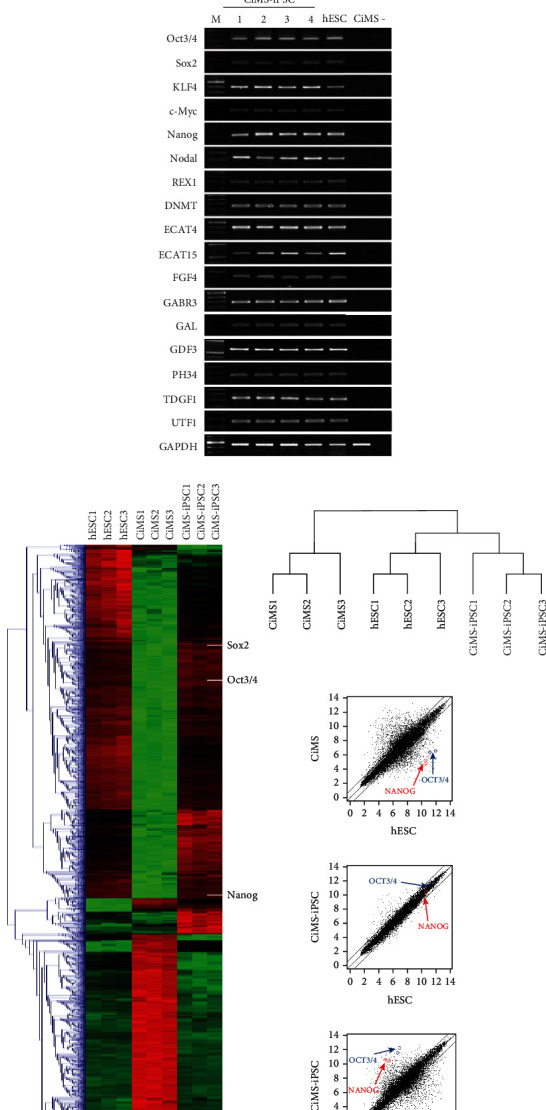
Generation of induced pluripotent stem cells (iPSCs) from human circulating multipotent adult stem (CiMS) cells. (a) Time schedule of CiMS-iPSC generation. (b) CiMS morphology before reprogramming factor transduction and human embryonic stem cell- (hESC-) like CiMS-iPSC colonies on feeder cells after transduction. (c) CiMS-iPSCs were positive for ALP staining and expressed pluripotency markers, such as OCT3/4, NANOG, and TRA-1-81. (d) Transduction efficiency was estimated using GFP retroviral transduction. Retroviral transduction efficiency was higher in CiMS than in human dermal fibroblasts (HDFs) and even 293T cells (*N* = 3) (^∗^*p* < 0.05, ^∗∗^*p* < 0.01, and ^∗∗∗^*p* < 0.001: statistically significant, ns: statistically not significant). (e) Comparison of ALP-positive colony formation efficiency of CiMS cells and HDF (*N* = 4) (f) Expression levels of CiMS-iPSC pluripotency genes were similar to those in hESCs, confirmed by reverse transcription PCR. (g) The global gene expression profiles compared CiMS, CiMS-iPSCs, and hESCs (H9) with the oligonucleotide microarray. (h) Bisulfite genomic sequencing of the promoter regions of OCT3/4 and NANOG. Open and closed circles indicate unmethylated and methylated CpGs. (i) Karyotyping analyses of the CiMS-iPSCs. (j) CiMS-iPSCs were spontaneously differentiated into three germ layers, and immunofluorescence staining showed positivity for each marker. The markers used were beta III tubulin and nestin for ectoderm, alpha-sarcomeric actin and smooth muscle actin for mesoderm, and AFP for endoderm. (k) Teratoma derived from CiMS-iPSCs. H&E staining of teratoma derived from CiMS-iPSCs. CiMS-iPSCs were transplanted subcutaneously on the back of a SCID mouse.

We isolated peripheral blood mononuclear cells and obtained CiMS cells using EGM-2MV with 95.6% efficiency within 2 weeks (Figure 1(a)). Then, 2 × 10^5^ CiMS cells were transduced with retroviral virus containing the genes described by Yamanaka (*Oct3/4*, *Sox2*, *KLF4*, and *c-Myc*) for 18 h. Five days after transduction, morphologically transformed cells started forming colonies (Figure 1(b)). Eight days after transduction, these transformed colonies were mechanically picked and passaged on the STO feeder layer (Figures 1(a) and 1(b)). CiMS-iPSCs were positive for ALP staining and expressed pluripotency markers such as Oct3/4, Nanog, and TRA-1-81 (Figure 1(c)). When we tested transduction efficiency with GFP retrovirus using 9.4 × 10^4^ TU virus, the transduction efficiency of CiMS cells was 93.75%, more than double that of human dermal fibroblasts (HDFs, 49.44%). The transduction efficiency was even higher than that of 293T cells (76.44%) (Figure 1(d)). We compared the efficiency of ALP-positive colony formation on a feeder layer after introducing a reprogramming factor into CiMS cells and HDFs. CiMS cells displayed approximately 1.47 times higher ALP-positive colony formation efficiency than HDF cells (Figure 1(e)). These results indicate that CiMS cells have higher potential for reprogramming into pluripotent stem cells than HDFs [16].

Reverse transcription PCR data showed the expression of pluripotent ES cell markers in CiMS-iPSCs. Various markers of human pluripotent ES cells (hESCs) were detected in all CiMS-iPSC clones at levels similar to those of hESCs. However, they did not appear in the parental CiMS cells (Figure 1(f)). To compare the global transcript profiles, cDNA of CiMS cells, hESCs, and CiMS-iPSCs were examined using DNA microarrays. Gene patterns upregulated in hESCs and CiMS-iPSCs appeared similar, but not with exactly the same pattern, and gene expression patterns in CiMS cells were very different from the previous two patterns. A total of 764,885 genes were analyzed by microarray in three different cell lines. Expression differences between CiMS cells and CiMS-iPSCs and between CiMS cells and hESCs showed large gaps, with 3,665 genes and 4,025 genes showing more than 2-fold difference in expression, respectively. In contrast, very few differences in expression appeared between CiMS-iPSCs and hESCs, with only 427 genes showing >2-fold difference (Figure 1(g)).

The methylation status of the CpG regions of the promoters of the pluripotent gene markers, *Nanog* and *Oct3/4*, was estimated by bisulfite genomic PCR (Figure 1(h)). The methylation percentages of CpGs in the *Oct3/4* promoter regions of CiMS cells, CiMS-iPSCs, and hESCs were evaluated as 40.8%, 3.3%, and 12%, respectively. The respective percentages for *Nanog* in CiMS cells, CiMS-iPSCs, and hESCs were 78%, 2.8%, and 7.1%, respectively. These results show that transcription of *Nanog* and *Oct3/4* is more active after transformation into iPSCs [8]. Karyotyping analyses of CiMS-iPSCs revealed a normal karyotype of 46XY (Figure 1(i)). To form embryoid bodies (EBs), CiMS-iPSCs floated in noncoated plastic dishes for 10 days. After 10 days, EBs were transferred to gelatin-coated culture dishes, EBs derived from CiMS-iPSCs were attached to the bottom of the dish, and differentiation was initiated. After 3 weeks, the cells were detected with *α*-fetoprotein (endoderm marker), *α*-sarcomeric actin (*α*-SA), *α*-smooth muscle actin (SMA) (mesoderm marker), nestin, GFAP, and *β*-III tubulin (ectoderm marker) using immunofluorescence (Figure 1(j)). To confirm the pluripotency of iPSCs *in vivo*, teratoma formation was examined [17]. Human CiMS-iPSC colonies were harvested and subcutaneously injected into nude mice. Two months after injection, encapsulated cystic teratomas were formed, and histological examination revealed that differentiated tissues from all three germ layers were present, including the endoderm (stratified squamous epithelium, mucus-producing epithelium, and ciliated epithelium), mesoderm (cartilage and skeletal muscle), and ectoderm (pigmented retinal epithelium and neuroepithelial rosette) (Figure 1(k)).

### 3.2. High Potential for CiMS-iPSC Differentiation into Cardiovascular Lineage Cells

**Figure 2:**
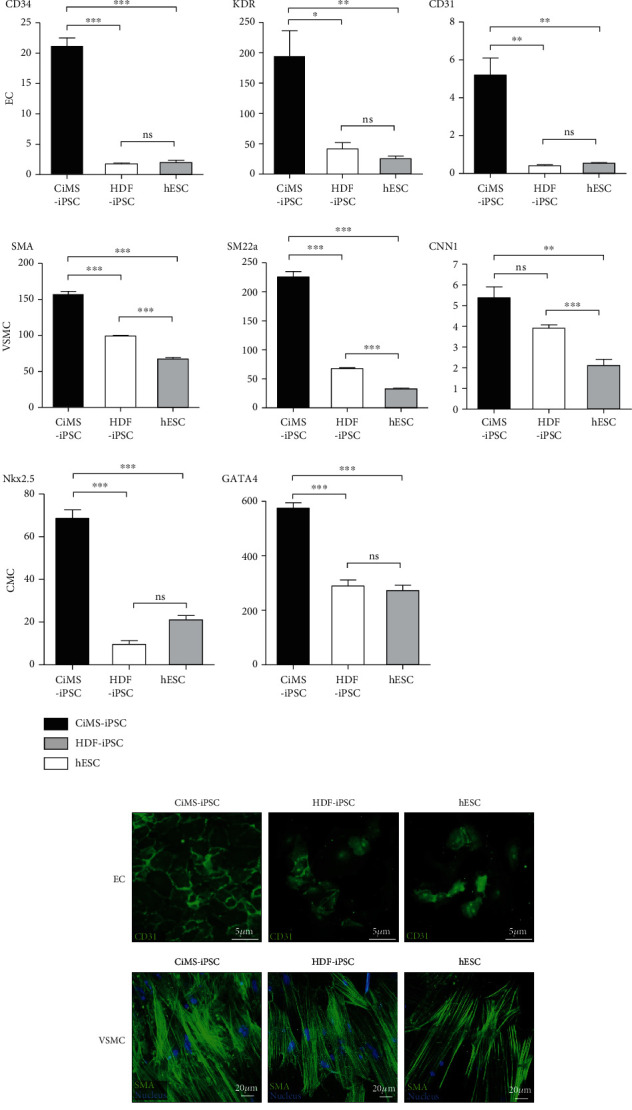
Mesoderm differentiation preference in the process of CiMS-iPSC differentiation to three germ layers. (a) Expression of pluripotent markers on day 0 and day 2 of mesodermal differentiation was estimated by real-time PCR. The expression of early mesodermal markers on day 2 of mesoderm differentiation was compared in each cell type using real-time PCR. The expression of early endodermal markers on day 4 of endodermal differentiation and early ectodermal markers on day 5 of ectodermal differentiation was compared in each cell type using real-time PCR (^∗^*p* < 0.05, ^∗∗^*p* < 0.01, and ^∗∗∗^*p* < 0.001; ss: statistically significant; ns: statistically not significant; black bar: CiMS-iPSC; white bar: HDF-iPSC; grey bar: hESC). (b) After differentiation into cardiovascular lineage cells, the expression of endothelial cell (EC) markers (CD34, KDR, and CD31) on day 5 of EC differentiation, vascular smooth muscle cell (VSMC) markers (SMA, SM22a, and CNN1) on day 6 of VSMC differentiation, and cardiomyocyte (CMC) markers (Nkx2.5, GATA4) on day 12 of CMC differentiation was compared in each cell type using real-time PCR. (c) After differentiation into cardiovascular cells, such as ECs, VSMCs, and CMCs, cardiovascular cell-specific markers were detected by immunofluorescent staining, including CD31 for ECs, SMA for VSMCs, and alpha-sarcomeric actin for CMCs. (d) Marker-positive cells were calculated among the total differentiated cells, and differentiation efficiency was compared.

There have been reports that the differentiation efficiency of iPSCs depends on their origin [6]. Since CiMS cells are cells found in the endocardium of the heart, we predicted that CiMS-iPSCs would be superior to iPSCs of other origins in differentiation into cardiovascular lineage cells. In order to observe the differentiation efficiency according to the epigenetic difference, iPSCs prepared from HDFs and CiMS cells and hESCs were compared. We treated iPSCs and hESCs with CHIR99021, a GSK3*β* inhibitor for mesodermal differentiation [18], and estimated the expression of mesodermal genes by real-time PCR. The pluripotency markers OCT3/4 and NANOG were downregulated in all iPSCs and hESCs upon initiation of differentiation (Figure 2(a)). As expected, CiMS-iPSCs showed increased expression of early mesoderm markers, compared to HDF-iPSCs or hESCs (Figure 2(a)). No such trend was observed in the differentiation of other germ layers, such as the endoderm or ectoderm (Figure 2(a)).

Next, we observed epigenetic differences in the differentiation into cardiovascular lineage cells, such as CMCs, endothelial cells (ECs), and vascular smooth muscle cells (VSMCs). When we differentiated each group cell into CMCs, ECs, and VSMCs, it was observed that each marker was strongly increased in CiMS-iPSCs, compared to HDF-iPSCs or hESCs (Figure 2(b)). Comparably, immunofluorescent staining for endothelial lineage markers, such as CD31; VSMC lineage markers, such as SMA; and CMC markers, such as alpha sarcomeric actin, showed that CiMS-iPSCs had higher potential to differentiate into those cardiovascular lineage cells than other cell types (Figures 2(c) and 2(d)).

### 3.3. High Mesodermal Differentiation Potential due to Different Epigenetic Status

**Figure 3:**
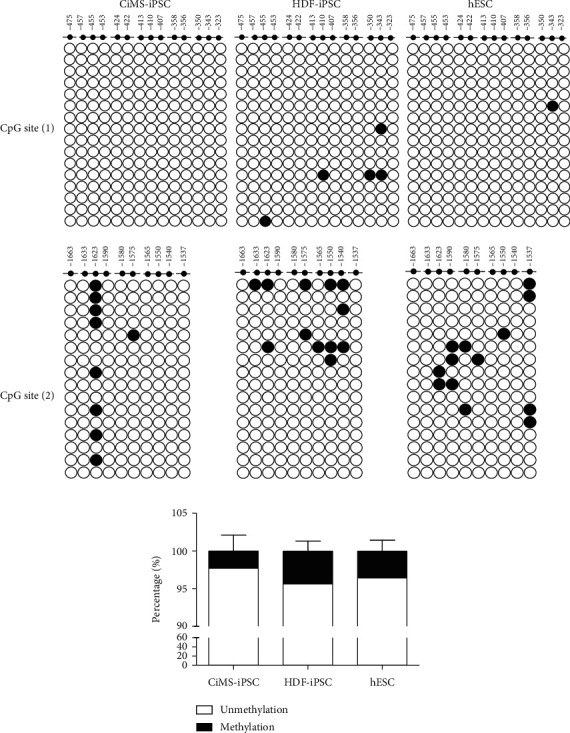
Comparison of brachyury T promoter methylation status in pluripotent cells. (a) Patterns of genomic DNA methylation at CpG site [1] (-475~-323) and CpG site [2] (-1537~1663) of brachyury T were confirmed by bisulfite PCR among the three different iPSCs. (b) Quantification of methylation status in the three cell types.

**Figure 4:**
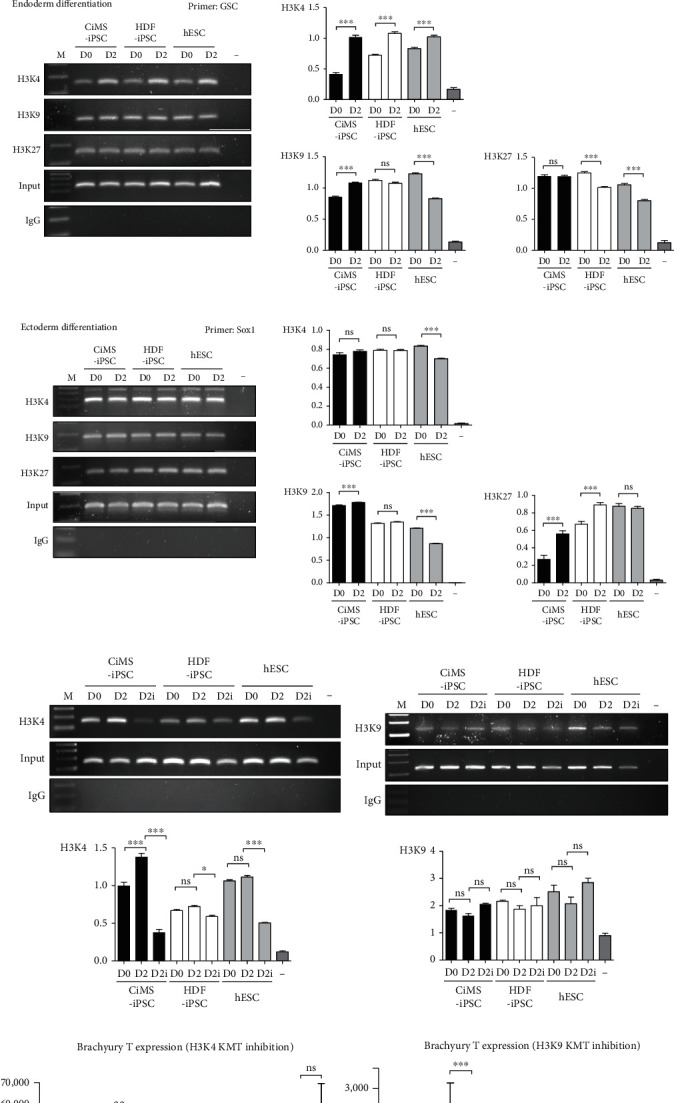
Comparison of histone tail modifications according to the three germline differentiation stages of each iPSC. (a) Histone tail modification levels of the transcription site of brachyury T during mesodermal differentiation (day 2 (D2)) of the three different cell types were compared using a chromatin immunoprecipitation (ChIP) assay. The H3K4me3, H3K9ac, and H3K27me3 levels of brachyury T were analyzed (^∗^*p* < 0.05, ^∗∗^*p* < 0.01, and ^∗∗∗^*p* < 0.001; ss: statistically significant; ns: statistically not significant). (b) Histone tail modification levels of the GSC transcription site during endodermal differentiation (day 2 (D2)) of the three different cell types were compared by ChIP assay. H3K4me3, H3K9ac, and H3K27me3 of GSC were analyzed. (c) Histone tail modification levels of the Sox1 transcription site during ectodermal differentiation (day 2 (D2)) of the three different cell types were compared by ChIP assay. H3K4me3, H3K9ac, and H3K27me3 of Sox1 were analyzed. (d) After treatment with the H3K4 KMT inhibitor (50 *μ*M MM102; D1i, D2i) during mesodermal differentiation (day 1 (D1), day 2 (D2)), H3K4me3 level of the brachyury T transcription site was compared in each cell type. (e) After treatment with the H3K9 KMT inhibitor (100 nM chaetocin; D1i, D2i) during mesodermal differentiation (day 1 (D1), day 2 (D2)), the H3K9ac level of brachyury T transcription site was compared in each cell type. (f) Brachyury T gene expression following H3K4 KTM inhibitor (MM102) treatment on mesoderm differentiation day 1 and day 2 was measured in each cell type using real-time PCR. (g) Brachyury T gene expression following H3K4 KTM inhibitor (chaetocin) treatment on mesoderm differentiation day 1 and day 2 was measured in each cell type using real-time PCR.

Epigenetic differences are mainly induced by methylation of gene promoters and modifications of histone proteins that cause changes in chromatin structure. To determine the cause of the tendency of CiMS-iPSCs to differentiate into cardiovascular cells, methylation of the CpG site of the brachyury T promoter, a mesoderm marker, was observed. When two CpG islands of the brachyury T promoter were examined using bisulfite PCR, there was little difference in CpG methylation between cells (Figures 3(a) and 3(b)). Therefore, we found that CpG methylation was not the cause of the differentiation tendency and observed histone modification of brachyury T through a chromatin immunoprecipitation (ChIP) assay. During mesoderm differentiation, H3K4me3 in the brachyury T promoter region was clearly increased in CiMS-iPSCs, and a tendency to decrease in H3K9ac levels in the brachyury T promoter region was observed. There was little difference in H3K27me3 in the brachyury T promoter region (Figure 4(a)). However, when iPSCs were differentiated into the endoderm (GSC) and ectoderm lineage (Sox1), there were no significant differences in histone modification levels between each cell group (Figures 4(b) and 4(c)).

A reverse experiment was conducted using the H3K4 transferase (KMT) inhibitor, MM102, and the H3K9 KMT inhibitor, chaetocin, in order to identify the role of histone modification more accurately. At the mesoderm differentiation stage, treatment with 50 *μ*M MM102 strongly suppressed H3K4me3 in the brachyury T promoter region of CiMS-iPSCs (Figure 4(d)) and reduced the expression of the brachyury T gene in CiMS-iPSCs (Figure 4(f)). In HDF-iPSCs, MM102 treatment had a slight inhibitory effect on H3K4me3 in the brachyury T promoter region, leading to a slight decrease in brachyury T gene expression (Figures 4(d) and 4(f)). In hESCs, H3K4me3 was suppressed, but brachyury T gene expression was not affected (Figures 4(d) and 4(f)). In contrast, treatment with 100 nM chaetocin slightly increased H3K9ac of the brachyury T promoter region (statistically not significant) (Figure 4(e)) but reversed the expression of brachyury T in CiMS-iPSCs (Figure 4(g)). In HDF-iPSCs and hESCs, chaetocin treatment had no effect on H3K9ac in the brachyury T promoter region, but the expression of brachyury T was reduced (Figures 4(e) and 4(g)). We performed additional experiments measuring the expression of cardiovascular lineage markers (Nkx2.5 and GATA4) using H3K4 KMT inhibitor or H3K9 KMT inhibitor (Supplemental Figure S1). Both inhibitors suppressed the expression of cardiovascular lineage markers.

### 3.4. Regenerative Potential of CiMS-iPSCs in Myocardial Infarction Model

**Figure 5:**
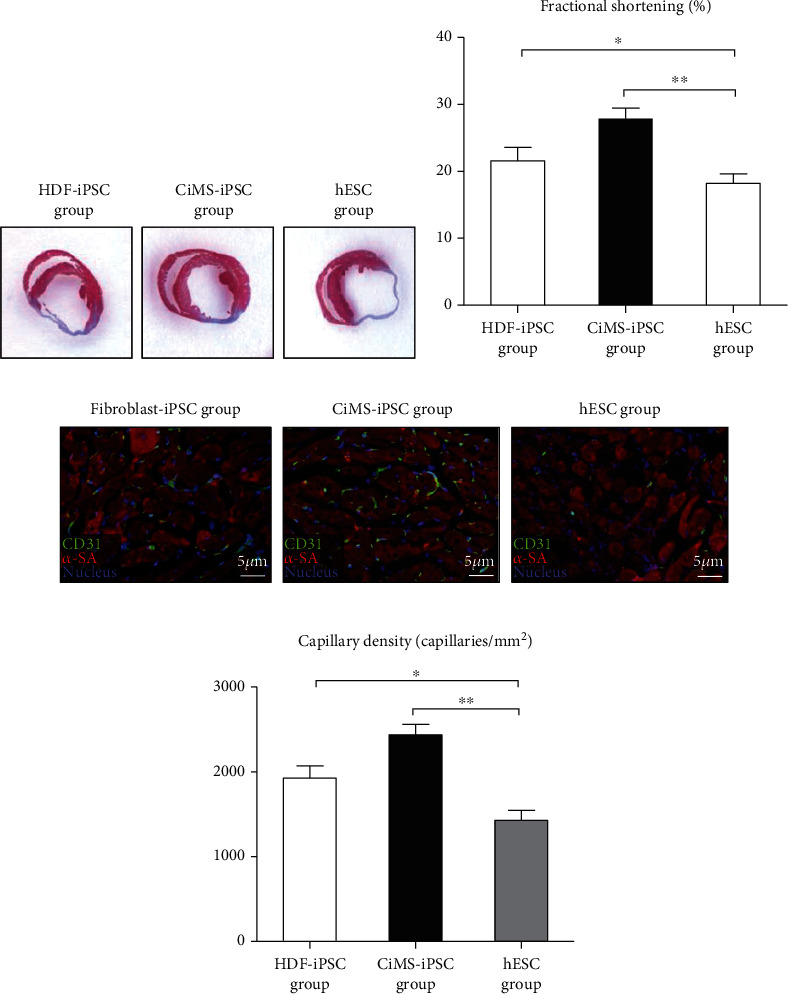
*In vivo* potential of CiMS-iPSCs in myocardial infarction model. (a, b) To check the *in vivo* therapeutic potential of CiMS-iPSCs, we performed a myocardial infarction model in nude mice. The group injected with differentiated CiMS-iPSC showed significant improvement in infarct area reduction and cardiac systolic function (mean ± SEM, HDF-iPSC vs. CiMS-iPSC vs. hESCs: 21.57 ± 2.1% vs. 27.90 ± 1.56% vs. 18.21 ± 1.39%, *p* value < 0.001; *n* = 10 per group). (c) CiMS-iPSCs could differentiate into endothelial lineage cells more efficiently than other groups. (d) Quantitative data of capillary density in each group.

To evaluate the *in vivo* therapeutic potential of CiMS-iPSCs, we employed a myocardial infarction model in nude mice. The group injected with CiMS-derived cells showed significant improvement in infarction area reduction and in heart systolic function (mean ± SEM, HDF-iPSC vs. CiMS-iPSC vs. hESC, 21.57 ± 2.1% vs. 27.90 ± 1.56% vs. 18.21 ± 1.39%, *p* value < 0.001; *n* = 10 per group) (Figures 5(a) and 5(b)). We also found that CiMS-iPSCs could differentiate into endothelial lineage cells more efficiently than the other cells (Figures 5(c) and 5(d)). In addition, we analyzed the difference in the number of the injected GFP-tagged cardiomyocytes in the border zone of each group (Supplementary Figure S2). We found that CiMS-iPSCs could differentiate into cardiomyocytes more efficiently than the other cells. These results suggest that the tendency of differentiation into cardiovascular lineage cells could contribute to therapeutic potential in regenerating damaged myocardium.

## 4. Discussion

**Figure 6:**
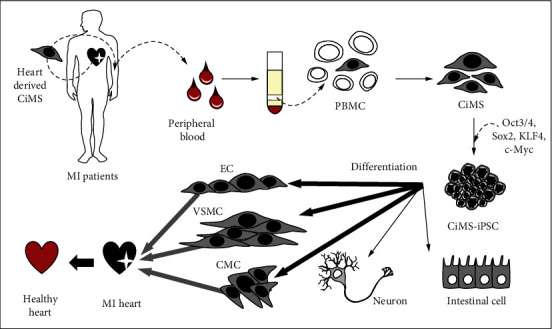
Production of iPSCs using CiMS cells and its application in cardiovascular cell differentiation. CiMS cells separated from the endocardium of the heart can be easily isolated from the blood and have high transduction efficiency; therefore, reprogramming is efficient. These CiMS cells are specialized for differentiation into cardiovascular cells even when they become iPSCs because of the epigenetic memory of the endocardium. Therefore, the generation of CiMS-iPSCs shows great advantages in cardiovascular disease-related modeling and is an appropriate cell line for stem cell therapy in regenerative medicine.

In this study, we presented CiMS cells, adult stem cells from the heart endocardium, a new candidate cell type for establishing iPSCs. CiMS cells can be easily obtained with a small volume of peripheral blood (10 ml) (Figure 6). This approach can prevent using invasive preparation methods for obtaining samples and long culture maintenance periods, which iPSC preparation from fibroblasts requires. CiMS cells showed a twofold higher transduction efficiency compared to fibroblasts (Figure 1(d)). These advantages make CiMS cells an ideal cell line for establishing iPSCs. After 14 days of culture, we introduced Yamanaka factors into CiMS cells and identified ES-like colonies without feeder cells (Figure 1(b)). These CiMS-iPSCs showed the essential features of iPSCs, including expression of pluripotent genes (Figures 1(f) and 1(g)), epigenetic status similar to hESCs (Figure 1(h)), *in vitro* differentiation ability into three germ layers (Figure 1(j)), and teratoma formation (Figure 1(k)).

CiMS-iPSCs differentiated more easily into the mesoderm than HDF-iPSCs and hESCs (Figure 2(a)). We have also shown that terminal differentiation into typical cardiovascular cells, such as ECs, VSMCs, and CMCs, was also observed with a more remarkable result in CiMS-iPSCs, compared to HDF-iPSCs and hESCs (Figures 2(b)–2(D)) [19–22]. Moreover, this tendency of CiMS-iPSCs resulted in improved regeneration of the infarcted myocardium (Figures 5(a) and 5(b)).

This tendency of CiMS-iPSCs to differentiate into cardiovascular lineage cells is presumed to be due to the epigenetic memory of CiMS cells. There have been reports that epigenetic features of the original cell can remain after reprogramming, which can affect their differentiation potential, even when iPSCs acquire the molecular and functional characteristics of hESCs [6, 7]. Recently, we confirmed that CiMS cells highly expressed the early CMC markers GATA4 and SOX17, which are involved in cardiac development [8]. Based on previous reports and our observations, we hypothesized that CiMS-iPSCs might be more potent in differentiating into cardiovascular cells than HDF-iPSCs and hESCs, as they present a more accessible epigenetic state than other iPSCs [8]. Genomic DNA methylation levels at the CpG site of brachyury T did not differ significantly among the three groups (Figure 3). However, we observed that the H3K4me3 level of the brachyury T promoter region in CiMS-iPSCs was significantly increased, and the H3K9ac and H3K27me3 levels of the brachyury T promoter region were decreased significantly during mesodermal differentiation (Figure 4(a)), whereas differentiation into ectoderm and endoderm showed no significant difference between PSCs (Figures 4(b) and 4(c)). Treatment with MM102, a H3K4 KMT inhibitor, strongly reversed H3K4me3 in the brachyury T promoter region of CiMS-iPSCs (Figure 4(d)) and reduced the expression of the brachyury T gene in CiMS-iPSCs (Figure 4(f)). In general, H3K4me3 is observed at gene promoters and functions as a genetically active signal [12]. In contrast, H3K27me3 and H3K9ac act as genetically repressive signals [13]. Therefore, the prediction that CiMS-iPSC could specialize in the differentiation of cardiovascular cells is appropriate when analyzing the results of histone methylation in the promoter region of brachyury T.

In conclusion, our study showed that the superior ability of CiMS-iPSCs to differentiate into cardiovascular lineage cells is due to their different epigenetics and histone modification status and not their DNA methylation status. CiMS cells are a feasible option for efficient iPSC generation. Moreover, our results suggest that their superior mesodermal differentiation ability could facilitate regenerative potential in various heart diseases, such as myocardial infarction.

## Data Availability

The data used to support the findings of this study are available from the corresponding author upon request.

## Supplementary Materials

Supplementary Materials The Supplementary Material for this article can be found online. Supplementary Figure S1: effect of H3K4 KMT inhibitor or H3K9 KMT inhibitor on the expression of cardiovascular lineage markers such as Nkx2.5 and GATA4. Supplementary Figure S2: differentiation potential of each group into cardiomyocytes in *in vivo* model. Supplementary Table S1: list of primers.

## References

[1] Takahashi K., Yamanaka S. (2006). Induction of pluripotent stem cells from mouse embryonic and adult fibroblast cultures by defined factors. *Cell*.

[2] Takahashi K., Tanabe K., Ohnuki M. (2007). Induction of pluripotent stem cells from adult human fibroblasts by defined factors. *Cell*.

[3] Yu J., Vodyanik M. A., Smuga-Otto K. (2007). Induced pluripotent stem cell lines derived from human somatic cells. *Science*.

[4] Park I. H., Lerou P. H., Zhao R., Huo H., Daley G. Q. (2008). Generation of human-induced pluripotent stem cells. *Nature Protocols*.

[5] Loh Y. H., Agarwal S., Park I. H. (2009). Generation of induced pluripotent stem cells from human blood. *Blood*.

[6] Kim K., Doi A., Wen B. (2010). Epigenetic memory in induced pluripotent stem cells. *Nature*.

[7] Kim K., Zhao R., Doi A. (2011). Donor cell type can influence the epigenome and differentiation potential of human induced pluripotent stem cells. *Nature Biotechnology*.

[8] Yang H. M., Kim J. Y., Cho H. J. (2020). NFATc1+CD31+CD45− circulating multipotent stem cells derived from human endocardium and their therapeutic potential. *Biomaterials*.

[9] Naveh-Many T., Cedar H. (1981). Active gene sequences are undermethylated. *Proceedings of the National Academy of Sciences of the United States of America*.

[10] Waechter D. E., Baserga R. (1982). Effect of methylation on expression of microinjected genes. *Proceedings of the National Academy of Sciences of the United States of America*.

[11] Strahl B. D., Allis C. D. (2000). The language of covalent histone modifications. *Nature*.

[12] Koch C. M., Andrews R. M., Flicek P. (2007). The landscape of histone modifications across 1% of the human genome in five human cell lines.

[13] Barski A., Cuddapah S., Cui K. (2007). High-resolution profiling of histone methylations in the human genome. *Cell*.

[14] Bernstein B. E., Mikkelsen T. S., Xie X. (2006). A bivalent chromatin structure marks key developmental genes in embryonic stem cells. *Cell*.

[15] Spivakov M., Fisher A. G. (2007). Epigenetic signatures of stem-cell identity. *Nature Reviews Genetics*.

[16] O'Connor M. D., Kardel M. D., Iosfina I. (2008). Alkaline phosphatase-positive colony formation is a sensitive, specific, and quantitative indicator of undifferentiated human embryonic stem cells. *Stem Cells*.

[17] Lensch M. W., Schlaeger T. M., Zon L. I., Daley G. Q. (2007). Teratoma formation assays with human embryonic stem cells: a rationale for one type of human-animal chimera. *Cell Stem Cell*.

[18] Tan J. Y., Sriram G., Rufaihah A. J., Neoh K. G., Cao T. (2013). Efficient derivation of lateral plate and paraxial mesoderm subtypes from human embryonic stem cells through GSKi-mediated differentiation. *Stem Cells and Development*.

[19] Coffin J. D., Poole T. J. (1988). Embryonic vascular development: immunohistochemical identification of the origin and subsequent morphogenesis of the major vessel primordia in quail embryos. *Development*.

[20] Pardanaud L., Dieterlen-Lievre (1999). Manipulation of the angiopoietic/hemangiopoietic commitment in the avian embryo. *Development*.

[21] Wasteson P., Johansson B. R., Jukkola T. (2008). Developmental origin of smooth muscle cells in the descending aorta in mice. *Development*.

[22] Wang G., Jacquet L., Karamariti E., Xu Q. (2015). Origin and differentiation of vascular smooth muscle cells. *The Journal of Physiology*.

