# Supplementary material for: Enhanced Generation of Human Induced Pluripotent Stem Cells from Peripheral Blood and Using Their Mesoderm Differentiation Ability to Regenerate Infarcted Myocardium
Source: Stem Cells Int. 2022 Feb 11;2022:4104622. doi: 10.1155/2022/4104622 (PMC8856835; doi:10.1155/2022/4104622)
Supplement: Supplementary Materials — The Supplementary Material for this article can be found online. Supplementary Figure S1: effect of H3K4 KMT inhibitor or H3K9 KMT inhibitor on the expression of cardiovascular lineage markers such as Nkx2.5 and GATA4. Supplementary Figure S2: differentiation potential of each group into cardiomyocytes in in vivo model. Supplementary Table S1: list of primers. [file 4104622.f1.docx]

**Supplementary Material**

**Efficient Generation of Induced Pluripotent Stem Cells from Human Peripheral Blood and Using Their Mesoderm Differentiation Ability to Regenerate Infarcted Myocardium**

Running Title: Blood-derived Stem Cells in MI

**Supplementary Figure S1.**

**
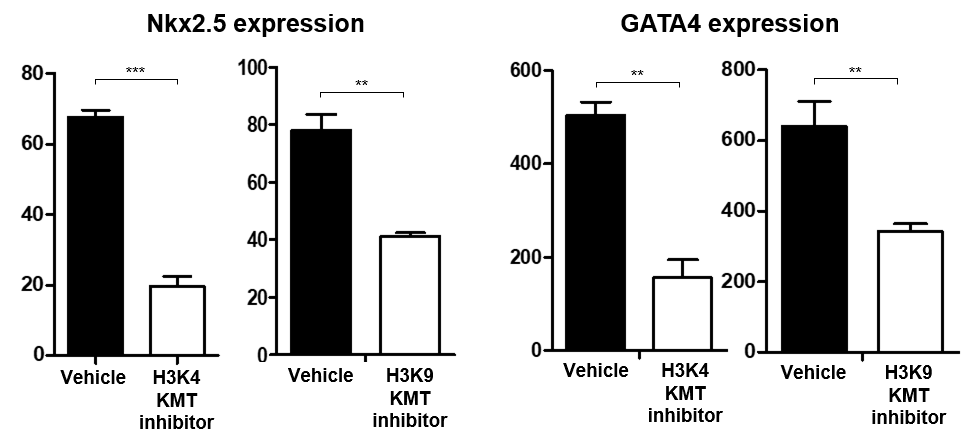
**

Effect of H3K4 KMT inhibitor or H3K9 KMT inhibitor on the expression of cardiovascular lineage markers such as Nkx2.5 and GATA4. (N=3~5) (*p <0.05, **p <0.01, ***p <0.001: statistically significant, ns: statistically not significant).

**Supplementary Figure S2.**

**
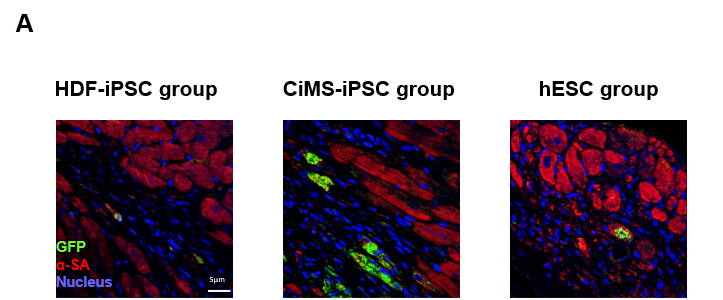
**

**
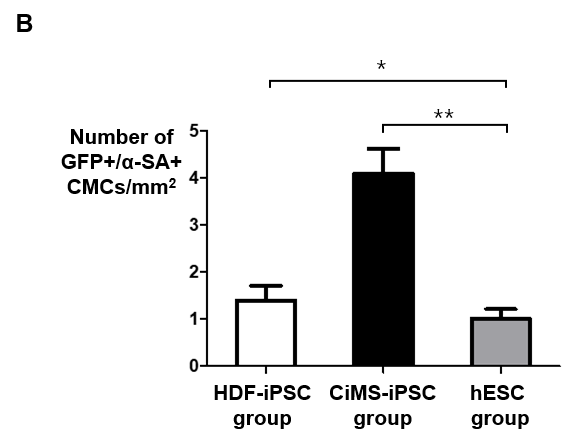
**

Differentiation potential of each group into cardiomyocytes in *in vivo* model.

(A) Images of the injected GFP-tagged cells of each group in the border zone in the myocardial infarction model. (B) Quantitative data of double positive cardiomyocytes for GFP and α-SA. (N=10) (*p <0.05, **p <0.01, ***p <0.001: statistically significant, ns: statistically not significant).

**Supplementary Table S1. List of primers**

| Gene name | F,R | Sequence (5’→3’) | Tm (℃) |
| --- | --- | --- | --- |
| Real time PCR-Mesoderm differentiation | | | |
| Mixl1 | F | TACCCCGACATCCACTTGCG | 60 |
|  | R | ATCTCCGGCCTAGCCAAAGG | 59 |
| Brachyury T | F | ACTGGATGAAGGCTCCCGTCTCCTT | 63 |
|  | R | CCAAGGCTGGACCAATTGTCATGGG | 63 |
| Wnt3a | F | ACTCCTCTGCAGCCTGAAGC | 56 |
|  | R | GAACTGGTGCTGGCACTCCT | 56 |
| CDH1 | F | GCATTGCCACATACACTCTC | 56 |
|  | R | AATCTCCATTGGATCCTCAA | 56 |
| Real time PCR-Ectoderm differentiation | | | |
| Nestin | F | AAGCCCTGAACCCTCTTTGC | 58 |
|  | R | GGGAAGAGGTGATGGAACCA | 58 |
| Sox1 | F | TCCCCCGCGTGAACTG | 54 |
|  | R | CAAGGCATTTTGCGTTCACA | 53 |
| Real time PCR-Endoderm differentiation | | | |
| GSC | F | GAGGAGAAAGTGGAGGTCTGGTT | 60 |
|  | R | CTCTGATGAGGACCGCTTCTG | 60 |
| Foxa2 | F | GGGAGCGGTGAAGATGGA | 57 |
|  | R | TCATGTTGCTCACGGAGGAGTA | 58 |
| Real time PCR-EC differentiation | | | |
| CD31 | F | TTCCTGACAGTCTCTTGAGTGGGT | 58 |
|  | R | TTTGGCTAGGCGTGGTTCTCATCT | 62 |
| KDR | F | GCGATGGCCTCTTCTGTAAG | 54 |
|  | R | ACACGACTCCATGTTGGTCA | 53 |
| CD34 | F | CCAGAGTTACCTACCCAGGG | 52 |
|  | R | TGGGGTGGTGAACACTGTGC | 59 |
| Real time PCR-VSMC differentiation | | | |
| SMA | F | CCAGCTATGTGTGAAGAAGAGG | 58 |
|  | R | GTGATCTCCTTCTGCATTCGGT | 58 |
| SM22a | F | CGCGAAGTGCAGTCCAAAATCG | 60 |
|  | R | GGGCTGGTTCTTCTTCAATGGGC | 62 |
| CNN1 | F | GAGTGTGCAGACGGAACTTCAGCC | 64 |
|  | R | GTCTGTGCCCAGCTTGGGGTC | 64 |
| Real time PCR-CMC differentiation | | | |
| Nkx2.5 | F | TTTGCATTCACTCCTGCGGA | 65 |
|  | R | ACTCATTGCACGCGTCATAATCGC | 64 |
| GATA4 | F | TCCAAACCAGAAAACGGAAG | 54 |
|  | R | TCGCACTGACTGAGAACGTC | 53 |
| Real time PCR-Housekeeping gene | | | |
| 18s rRNA | F | CCTGCGGCTTAATTTGACTC | 56 |
|  | R | ACCAACTAAGAAGAACGGCATG | 57 |
| ChIP assay | | | |
| Brachyrury T | F | GAAAGCAATGACACAGCAGA | 56 |
|  | R | AGGGAAATGGACGGAAATAA | 55 |
| SOX1 | F | GCGAGGAGACAGCACACC | 59 |
|  | R | CCTGATGCACAAACCACTTG | 56 |
| GSC | F | GAGCTACAGGCAGAGGAAATCGCA | 62 |
|  | R | CTGGGCGGGCGGCCTAATTG | 62 |
